# The contribution of family physicians and primary care doctors to community-orientated primary care

**DOI:** 10.4102/safp.v63i1.5281

**Published:** 2021-03-10

**Authors:** Robert Mash, Bernhard Gaede, Johannes F. Hugo

**Affiliations:** 1 Division of Family Medicine and Primary Care, Faculty of Medicine and Health Sciences, Stellenbosch University, Cape Town, South Africa; 2 Department of Family Medicine, Nelson R Mandela School of Medicine, University of KwaZulu-Natal, Durban, South Africa; 3 Department of Family Medicine, University of Pretoria, Pretoria, South Africa

**Keywords:** primary healthcare, primary care, community orientated primary care, population health management, family physicians, general practitioners, primary care doctors

## Abstract

South Africa envisages a community-orientated approach to primary health care (PHC). Family physicians and primary care doctors have important roles to play in leading, implementing, supporting and maintaining community-orientated primary care (COPC). In this article, we define COPC, its key principles and approaches to implementing it in health services. Following this we describe the key competencies expected of family physicians and primary care doctors in leading and supporting its implementation; providing clinical support to the PHC teams and linking these teams to other parts of the health system, other sectors and the community. The required knowledge and skills underlying these competencies are also discussed and some specific tools included.

## Introduction

The primary health care (PHC) system in South Africa is organised around a commitment to community-orientated primary care (COPC).^1^ This means that family physicians and primary care doctors have important roles to play in leading, implementing, supporting and maintaining COPC. For many doctors this will require some re-orientation and development of new competencies.

The move towards COPC was driven by a desire to address the health needs of the whole population and not just those that access primary care facilities, with an emphasis on health promotion and disease prevention for both individuals and communities.^1^ South Africa invented the concept of COPC in the 1940s and exported it around the world.^2^ In many ways COPC embraces the core principles of a PHC approach as described in the Astana Declaration.^3^ Such an approach can be very cost-effective with the resources available in South Africa.^4^ The PHC performance initiative also describes population health management and primary care as key aspects of effective service delivery.^5^

This commitment to a COPC approach is seen practically through the widespread implementation of ward-based outreach teams (WBOT).^1^ This means that each geographically defined ward or similarly delineated community has a team of community health workers (CHWs), led by a nurse and supported by a primary care doctor. As we move forward to the implementation of national health insurance, each contracting unit for PHC will need to accredit both community-based and facility-based services in a COPC framework.^6^ At this point all practitioners, whether in the public or private sector, will need to understand COPC.

## What is community-orientated primary care?

Community orientated primary care has been defined as a continuous process by which PHC is provided to a defined community on the basis of its assessed health needs, by the planned integration of primary care practice and public health.^7^

The need for this type of integration has also been highlighted by the response to the coronavirus pandemic – where PHC teams were instrumental in home delivery of medication,^8^ community screening and testing^9^ as well as in the future vaccinations. It differs from other approaches to healthcare delivery in that it explicitly makes the link between clinical services to individuals with broader interventions at the community level. Community interventions may be in household, working, learning or social spaces.

Nine key principles of COPC have been identified in the African region (Table 1).^10^

**TABLE 1:** Principles of community-orientated primary care.

Principle	Definition
A defined community	The community served is specifically defined, usually in geographic terms.
A multidisciplinary team approach	COPC involves a team of health workers; typically community health workers, nurses and sometimes doctors.
A comprehensive approach	Within the defined community a COPC approach engages people of all ages, genders and includes attention to health promotion, disease prevention, care, rehabilitation and palliation.
An equitable approach	COPC should be accessible, appropriate, affordable and relevant to everyone in the community. Health equity may be improved.
Analysis of local health needs and assets	COPC includes assessment of the health needs of the community as well as the inter-sectoral resources available to assist with these needs
Prioritisation of health needs and interventions	The analysis of health needs leads to a process of prioritisation and then development of interventions to address these priorities that involve stakeholders from different sectors.
Community participation	The analysis of health needs, prioritisation, planning and action should be done in a participatory approach with community members or structures.
Evidence-based and scientific	COPC uses data collected from households, facilities, research and other sources to identify and respond to individual, household and community health needs.
Service integration around users	COPC is fundamentally person-centred in how services are coordinated and continuous.

Source: Mash R, Ray S, Essuman A, Burgueño E. Community orientated primary care in the sub-Saharan Africa context: A scoping review of different models, their effectiveness and feasibility. Br Med J Glob Health. 2019;4:e001489. https://doi.org/10.1136/bmjgh-2019-001489

COPC, community-orientated primary care.

**FIGURE 1:**
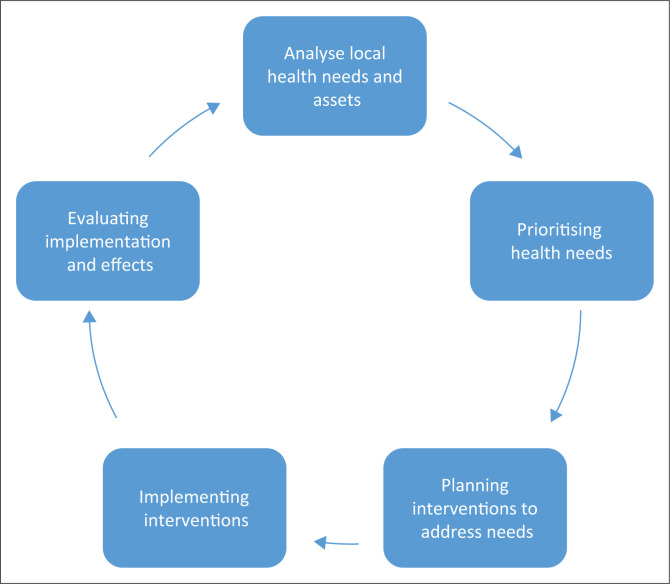
The community-orientated primary care cycle.

The process of analysing local health needs and assets, prioritising those needs, planning interventions and evaluating them can be seen as a COPC cycle (Figure 1). Community and stakeholder participation in this process is also essential. In the African context COPC most often involves CHWs as illustrated by our WBOTs.

**FIGURE 2:**
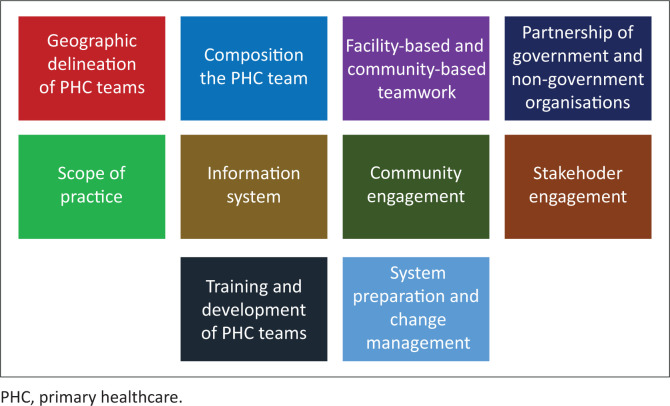
Elements of the framework to implement community-orientated primary care in Metro Health Services, Cape Town.

The practical implementation of COPC has been articulated in a variety of settings locally and internationally.^10^ It has been used in both high- and low-income countries, in rural and urban communities and at times with interventions that have a particular focus such as homeless people, or follow-up post cardiac surgery.^10,11^ In South Africa large projects are being implemented amongst others in Gauteng and Cape Town. In Cape Town, for example, a 10-point framework for implementation of COPC has been adopted as shown in Figure 2 and Table 2.^12^ A number of training manuals on how to implement COPC have come out of the Tshwane experience^13,14,15^ as well as a special collection of research studies.^16^

**TABLE 2:** The Cape Town framework for implementation of community-orientated primary care.

Key element in framework	Description
Geographic delineation of PHC teams	The community served by a primary care facility is defined and delineated into a series of contiguous areas that are served by a PHC team.
Composition of the PHC team	The core PHC team consists of 10–15 community health workers (CHWs), a professional nurse, supported by a clinical nurse practitioner and primary care doctor. Each CHW is responsible for approximately 250 households or 1000 people.
Facility-based and community-based teamwork	The primary care providers in the facility and community-based members of the PHC team must form one integrated functional team.
Partnership of government and non-government organisations	In Cape Town, the CHWs and professional nurses are employed via non-government organisations (NGOs). Ensuring an effective partnership between these NGOs and governmental health services is vital. In other parts of the country everyone is employed directly by the government.
Scope of practice	The scope of practice of each member of the team needs to be clearly defined. The community-based team members can work in living (e.g. households), social (e.g. religious institutions), working (e.g. local businesses) or learning spaces (e.g. schools) within the delineated community. The scope of practice is comprehensive across the life course and involves health promotion, disease prevention, treatment, rehabilitation and palliative care.
Information system	The information system should integrate information from community-based and facility-based settings for both individuals and the community as a whole. M-health technology is particularly suited to CHWs.
Community engagement	Health services should pursue both formal (e.g. clinic health committees) and informal (e.g. local health forums) ways of engaging with the community and ensuring their understanding and participation.
Stakeholder engagement	Health services should engage actively with other stakeholders contributing to health in the community, for example, other primary care providers and practitioners, public and private, alternative and traditional. Inter-sectoral engagement and collaboration is also important. For example, with social services, educational services, neighbourhood watches and police.
Training and development of PHC teams	All members of the PHC team will need training commensurate with their roles in a COPC approach. For example, CHWs will need to be trained in a generalist and comprehensive approach as many were originally focused on more targeted programmes. Nurses will need training in how to work in communities as well as supervise and support CHWs. Training may be a blend of more formal classroom based teaching and ongoing experiential learning.
System preparation and change management	COPC requires adequate inputs to work effectively in terms of funding, resources, supplies, transport, infrastructure, workforce and information. Managers and leaders at all levels of the system need to have the same understanding and support the development of COPC. Policies need to be aligned with the COPC approach. The COPC approach needs to be communicated and explained to the public.

Source: Mash R, Goliath C, Mahomed H, Reid S, Hellenberg D, Perez G. A framework for implementation of community orientated primary care in the Metro Health Services, Cape Town, South Africa. Afr J Prm Health Care Fam Med. 2020;12(1), a2632. https://doi.org/10.4102/phcfm.v12i1.2632

PHC, primary healthcare; COPC, community-orientated primary care.

## Roles of the family physician and primary care doctor

Many of the roles and competencies around COPC stretch beyond the clinical role. However, this does not negate the need for high-quality clinical services to be provided and supported. In fact, the provision of a clinical service offers powerful opportunities to transform and build stronger links with health promotion and disease prevention strategies at the household and community levels.^17^

### Lead and support the implementation of community-orientated primary care

The current policy direction is supportive of developing COPC approaches at primary care facilities. Family physicians are often located in districts, district hospitals, sub-districts and community health centres where they can help lead the implementation of COPC with local decision makers and implementers. Primary care doctors are also in a position to help with this for the community they serve from their facility or practice. Regardless of where they are based, their responsibility stretches beyond the walls of the institution.

Practically speaking this may involve the following activities:

- Explaining the principles of COPC to other healthcare workers and managers
- Collaborating with the local management team to plan and implement COPC
- Assisting with the interpretation of data on the community’s health assets and health needs.^18^
- Assisting with the prioritisation of health issues in the community.^18^
- Assisting with the planning of responses or interventions to address these health issues in the community.
- Participating in the implementation of these responses or interventions in the community.
- Assisting with the evaluation of these responses or interventions.
- Advocating for vulnerable and neglected individuals, or communities within the population served by the healthcare team.

**FIGURE 3:**
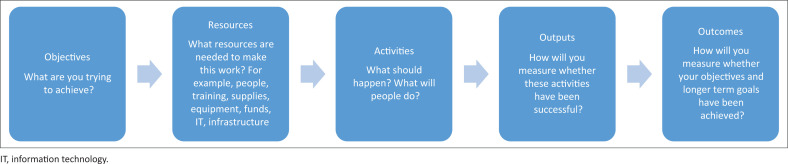
A logic model approach.

Underlying these competencies is a need to understand and embody COPC principles, to build relationships with key role players, to analyse and interpret data, to facilitate prioritisation processes such as the nominal group technique (see Box 1), to facilitate rational planning processes and design of interventions as well as plan their evaluation. Design of projects may require the ability to clarify the logic behind the project that is expected to lead to change (see Figure 3). Such a logic model can be used to plan evaluation of the intervention. Implementation science may be of particular use with a mixed-methods approach to evaluating a range of implementation outcomes (see Box 2).^19^ Mixed methods implies the collection of both quantitative data (e.g. routinely collected, a simple survey) as well as qualitative data (e.g. interviews) to evaluate implementation. In addition there will be measures of the expected effects or effectiveness (outputs and outcomes) that need to be collected, although these may take longer to change.

BOX 1 Nominal group technique for prioritisation of ideas.

Step 1 – Silent phase: Give each member of the group a piece of A4 paper on which is written the specific question that you want them to think about. Ask them to write down as many ideas or responses as they can to the question. Each person should work alone and in silence for 15–20 min.

Step 2 – Item generation, or round-robin phase: Form people into sub-groups of five or six and have each one elect a scribe. Each person then reads out one idea/response, which the scribe records on a flip chart, until all the ideas are recorded. This ensures equal participation from everyone. No discussion or comment on the ideas is allowed at this stage.

Step 3 – Item clarification phase: Each sub-group discusses the items recorded to ensure that the meaning is clear and shared by all. They can combine or edit items that duplicate or overlap, but they should not discard any items. At the end of this process, they should create a clearer and shorter list of items.

Step 4 – Voting phase: Each person must choose five items from the list that are most important to him and rank them in order of priority on a scale of 5 (most important) to 1 (least important). They write down their selection and ranking on a voting paper, all of which are then collected. Compile all the prioritised items from each sub-group into a master list, although not in rank order.

Step 5 – Reassembly of group phase: The entire group now repeats steps 3 and 4 for the master list. Collect the final voting papers and analyse them to give a final ranking of the items that the group has prioritised.

Source: Buso D, Reid S. How to make a community diagnosis and prioritise health issues. In: Mash B, Blitz J, editors. South African family practice manual. 3rd ed. Pretoria: Van Schaik, 2015; p. 497–500.

Box 2 Implementation outcomes.

*Acceptability of the intervention:* Do stakeholders perceive that it is worth doing? What are the factors for and against this? How does it align with other goals and policies?

*Adoption of the intervention:* Do stakeholders decide to collaborate and adopt the intervention? What are the key factors they considered in making this decision?

*Appropriateness of the intervention:* Do stakeholders perceive that the intervention is fit for purpose and relevant? Which aspects are a better fit, or a worse fit?

*Feasibility of the intervention:* What happens, when is it implemented? How feasible is it to implement it successfully? What are the factors that influence implementation?

*Fidelity of the intervention:* How is the intervention modified or customised to make it work? Why was this necessary?

*Coverage of the intervention:* How many people were reached? Who was reached? Who was not reached?

*Costs of the intervention:* What were the set-up costs? What were the additional costs? How were existing costs allocated to this intervention (e.g. time of employees)

*Sustainability of the intervention:* What are the future opportunities and threats to the sustainability of the intervention? Should it continue? How can it be scaled?

Source: Adapted from Peters DH, Adam T, Alonge O, Agyepong IA, Tran N. Implementation research: What it is and how to do it. Br Med J [serial online]. 2013 [cited 2019 Apr 5];347:f6753. Available from: https://www.bmj.com/content/347/bmj.f6753

### Provide support to the primary healthcare teams in your community

As a clinician you will be used to providing support to more junior doctors, nurse practitioners and others in your facility. With a COPC approach such support extends to the community-based nurses and CHWs. Community health workers may refer patients to you for help in the facility or in the community. At times this may necessitate a home visit to support the PHC team if, for example, the patient is complicated and housebound.^20^

Family physicians usually take responsibility for leading clinical governance along with the rest of the team.^21^ Clinical governance focuses on the quality of care and patient safety. In a COPC approach, this responsibility extends to the whole PHC team and not just care in the facility. It therefore includes the clinical governance processes for the care in the home, the quality of the referral system and the inclusion of services such as rehabilitation or palliative care. Clinical governance activities may include:

- Training of other healthcare workers
  ■ For example, training CHWs in use of personal protective equipment or screening for the coronavirus disease 2019 (COVID-19), responding to problems or challenges that arise from practice
- Clinical audit and feedback
  ■ For example, quality of active surveillance for tuberculosis (TB) in the community^22^
- Facilitating reflection on health information
  ■ For example, the analysis of household assessment data collected by CHWs^23^
- Implementation of new guidelines
  ■ For example, how CHWs can calculate cardiovascular risk with non-laboratory data^24^
- Review and appraise new evidence for the team
  ■ For example, value of home delivery of medication by CHWs^25^
- Participating in research to address questions that arise from practice
  ■ For example, what is the role of CHWs in non-communicable diseases

These activities also imply a wide range of underlying knowledge, skills and attitudes. Creating a learning environment is important to continuously improve and to train or teach effectively.^26^ The ability to analyse and interpret data, appraise evidence and sometimes create new evidence is also key.

### Link the primary healthcare teams to the rest of the health system, other sectors and community

The family physician and primary care doctor should pay particular attention to the coordination of care within and between PHC teams. For example, help in building a strong relationship between facility-based and community-based members of the PHC team, to coordinate care for the patients. The introduction of home delivery of medication by CHWs during COVID-19, for example, necessitated much closer cooperation between clinicians, pharmacists, nurses and CHWs.^8^

Family physicians and doctors may also be a strong link between the PHC team and the local district hospital, or higher levels of care. Multidisciplinary ward-rounds can include members of the PHC team and improve coordination of care for patients as well as improve the understanding of the hospital teams in the context of the community. It is important that each hospital, health centre and clinic has a specific plan to link COPC practice to care in the institution. This is in the form of care-coordination ward rounds, multidisciplinary patient and family discussions, well-structured referral letters, discharge summaries and patient retained records. In some hospitals a team of CHWs and a WBOT team leader works full time in the hospital to facilitate the coordination of care. This should be facilitated and supported by the family physicians and other doctors.

Family physicians and doctors should also use their leadership positions to build relationships with other sectors, particularly social services. Health and social services often go hand-in-hand and require mutual linkages and collaboration. Other sectors may be important depending on the prioritised needs of the community. For example, concern for illegal circumcision schools or crime and violence may need links to the police and neighbourhood watch, whilst concern for teenage pregnancy may need links to the educational sector. As mentioned above, this often involves a range of advocacy skills.^27^

Family physicians and doctors should also use their leadership positions and authority to build relationships with formal and informal community structures. Participating in local community health forums may enable community engagement and participation in the COPC cycle.

Whilst such coordination and relationship building is not the sole responsibility of the family physician or other doctors, they are in a powerful position to offer leadership alongside managers and senior health professionals.

## Conclusion

South Africa has moved towards a COPC approach to providing health services, which has tremendous potential for improving the health of communities. Family physicians and primary care doctors need to understand and embody the principles of this COPC approach so that they can help lead the health services in this direction. They will also need to provide clinical support and clinical governance to the whole PHC team engaged in COPC. They can also be instrumental in coordinating care within and between levels of the health system, supporting community and stakeholder engagement. These various roles and activities require an expanded knowledge base and skills-set, beyond the traditional clinical roles.
